# Complex Liver Resections for Intrahepatic Cholangiocarcinoma

**DOI:** 10.3390/jcm10081672

**Published:** 2021-04-13

**Authors:** Tim Reese, Gregor Pagel, Bettina A. Bause, York von Rittberg, Kim C. Wagner, Karl J. Oldhafer

**Affiliations:** 1 Asklepios Campus Hamburg, Semmelweis University of Medicine, 20099 Hamburg, Germany; k.oldhafer@asklepios.com; 2 Department of Surgery, Division of Hepatobiliary and Pancreatic Surgery, Asklepios Hospital Barmbek, Rübenkamp 220, 22291 Hamburg, Germany; p.gregor@asklepios.com (G.P.); b.bause@asklepios.com (B.A.B.); y.rittberg@asklepios.com (Y.v.R.); ki.wagner@asklepios.com (K.C.W.)

**Keywords:** liver resection, cholangiocarcinoma, vascular reconstruction, UICC, ante situm

## Abstract

The only curative treatment option for intrahepatic cholangiocarcinoma (iCCA) is liver resection. Due to central tumor localization and vascular invasion, complex liver resections play an important role in curative treatment. However, the long-term outcomes after complex liver resection are not known. **Methods:** A retrospective cohort study was conducted for all patients undergoing liver surgery for iCCA. Complex liver resections included ante situm resections, associating liver partition and portal vein ligation for staged hepatectomy (ALPPS) and major liver resection with vascular reconstructions. **Results:** Forty-nine patients (34%) received complex liver resection, 66 patients (46%) received conventional liver resection and 28 patients (20%) were not resectable during exploration. Preoperative characteristics were not different between the groups, except for Union for International Cancer Control (UICC) stages. The postoperative course for complex liver resections was associated with more complications and perioperative mortality. However, long-term survival was not different between complex and conventional resections. Independent risk factors for survival were R0 resections and UICC stage. Four patients underwent ante situm resection without any mortality. **Conclusions:** Complex liver resections are justified in selected patients and survival is comparable with conventional liver resections. Survival in iCCA is affected by UICC stage or resections margins and not by the complexity of the case.

## 1. Introduction

The incidence of intrahepatic cholangiocarcinoma (iCCA) has increased over recent decades [1,2,3]. This might be due to improvements in diagnostic tools or increases in metabolic disorders and obesity [4]. Advances in local therapies, targeted chemotherapies and adjuvant chemotherapy are promising [5,6,7,8]; however, liver resection remains the only potential curative therapy [9,10]. At presentation, about 50% of patients have unresectable disease and another 30% will be found unresectable during surgical exploration [11]. Therefore, the resectability rate of iCCA is described as 15–35% [11,12,13]. When the tumor is confined to the liver, complete tumor resection results in a 5-year survival rate of up to 40% [11,13,14,15]. The survival and recurrence rates are affected by nodal metastasis, tumor size, multifocal tumor growth and macrovascular invasion [12,16]. Central localization of iCCA is quite common and vascular invasion of the hepatic veins is often a limiting factor for R0-resection. Regarding these circumstances, non-conventional surgical techniques, such as ante situm procedures, offer a possibility for resection [17,18,19,20,21]. In addition, portal vein resection is often needed and complex constructions are described as safe for selected patients in high-volume centers [9,22,23].

A sufficient future liver remnant (FLR) is critical in decreasing postoperative morbidity and mortality [24,25]. Tumor size and location represent important factors for the resection strategy. Not seldom, complex liver resections such as meso-hepatectomy or trisectionectomy with reconstruction of the extrahepatic biliary duct are necessary [12,26].

In case of a non-sufficient FLR, portal vein embolization (PVE) or associating liver partition and portal vein ligation for staged hepatectomy (ALPPS) are strategies to enhance FLR [12]. Whereas PVE is considered as safe and standard technique for small FLR [9,27], the role of ALPPS is currently controversially discussed [28,29,30].

However, in liver resection for iCCA, the main focus is to accomplish R0-resection, even with the use of complex surgical techniques, such as ante situm or extended resections with or without vascular reconstructions, if necessary. The aim of this study is to analyze the role of complex liver resections for iCCA in terms of morbidity and long-term survival.

## 2. Methods

### 2.1. Patient Selection and Definitions

Consecutive patients that underwent surgery for iCCA between January 2010 and December 2020 at the Division of Hepatobiliary and Pancreatic Surgery in the Asklepios Hospital Barmbek were included in this retrospective study. Data were obtained from a prospectively collected database.

All included patients were divided into three groups:

(a) Conventional liver resections: major anatomical or non-anatomical hepatectomy without vascular reconstruction and any minor anatomical or non-anatomical hepatectomy.
(b) Complex liver resections: extended liver resection or major anatomical hepatectomy with vascular reconstruction, ALPPS and ante situm resection.
(c) Exploration: Patients were preoperative, considered as resectable but were found to be unresectable during exploration.

Lymphadenectomy was routinely performed in all major liver resections. For minor liver resection, a lymphadenectomy was performed if there was a preoperative or intraoperative suspicion of positive lymph nodes. All complications were graded according to the Clavien–Dindo Classification [31] and summarized by the Comprehensive Complications Index (CCI) [32]. The Charlson Comorbidity Index was calculated as previously described [33]. All liver resections were defined according to the Brisbane 2000 Terminology [34]. Post-hepatectomy liver failure (PHLF) was defined according to the International Study Group of Liver Surgery (ISGLS) criteria [35].

### 2.2. Statistical Analysis

Data are reported as median with interquartile range (IQR), and *n* with percentages (%), where appropriate. Continuous variables were compared with the Mann–Whitney U and Kruskal–Wallis tests, where appropriate. Differences among proportions from categorical data were compared using the Fisher’s exact or the Pearson v2 tests, where appropriate. Survival analysis was performed using Kaplan–Meier curves. Patients lost to follow-up or follow-up time ended were censored. Differences in survival were compared using the log-rank test. All *p*-values in the univariate analysis were 2-sided and considered statistically significant if *p* ≤ 0.05. Cox regression analysis was performed to identify independent predictors of overall survival. Statistical analysis was performed using SPSS, version 27 for Windows (IBM, Armonk, NY, USA).

## 3. Results

### 3.1. Study Population

Out of 1906 liver surgeries during the study period, 143 patients underwent surgery because of iCCA. Forty-nine patients (34%) received complex liver resection, including four ante situm resections, seven ALPPS, 10 extended left and 19 extended right hepatectomies and nine major hepatectomies with vascular reconstruction (three right hemi-hepatectomies, three left hemi-hepatectomies and three other anatomical resections). Vascular reconstructions consisted of five interpositions of the inferior vena cava (IVC) with a vascular graft, two patch reconstructions of the IVC, eight resections and reconstructions of the portal vein and one patch reconstruction of the portal vein. Conventional liver resection consisted of 66 patients (46%), including 17 non-anatomical resections, 16 right hemi-hepatectomies, 9 left hemi-hepatectomies, 11 bi-segmentectomies and 13 segmentectomies. Twenty-eight patients (20%) were not resectable during exploration.

### 3.2. Preoperative Characteristics

No differences could be seen between the groups regarding age, gender, and comorbidities (Table 1). Union for International Cancer Control (UICC) stages were significant different between the groups. Stage IV was highest in the complex (51%) and exploration group (82%), but only 29% for conventional resections. With 64%, the early stages (Stage I and II) were most common for conventional resections, whereas it was low for complex (37%) and exploration (18%).

### 3.3. Operative and Resection Details

Operation time, incidence of hepaticojejunostomies, use of intraoperative transfusions and blood loss were significantly highest for the complex group (Table 2). The use of pringle maneuver was significant, but comparable between complex and conventional resections. R0-resection was achieved in 63% of patients undergoing complex resections and 86% for conventional resections.

**Figure 1:**
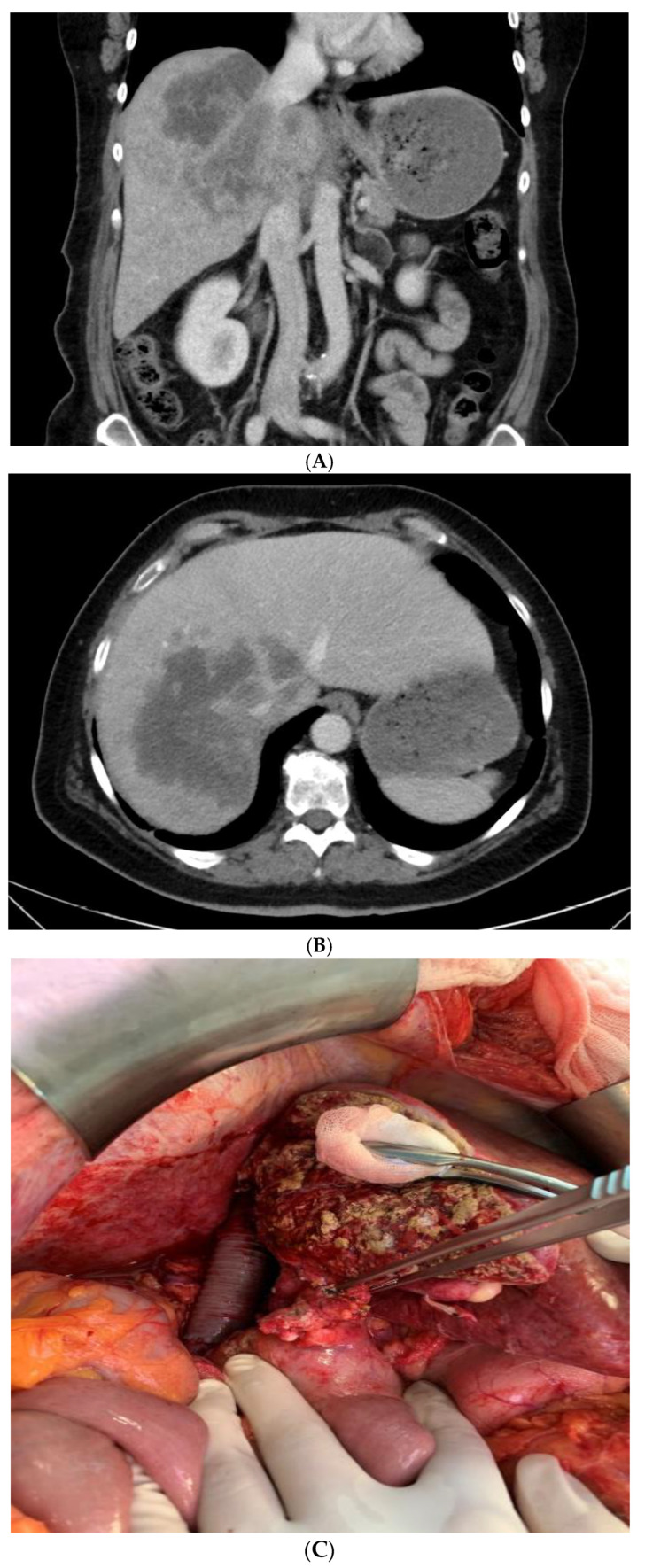
A 55-year-old female patient with cholangiocarcinoma and nodular metastases (cT2 cN1 cM1 (LYM)) received neoadjuvant chemotherapy with Gemcitabine and Cisplatin. Preoperative CT scan showed a stable disease with tumor in the right liver lobe (**A**) and infiltration of the right and middle hepatic vein (**B**). To augment the future liver remnant, a portal vein embolization was carried out prior the planned major hepatectomy. We performed a trisectionectomy (Segment I, IV–VIII) combined with replacement of the inferior vena cava and reinsertion of the left hepatic vein (**C**), using femoral-axillary bypass and portal hypothermic liver perfusion. A R0-resection was histopathological secured.

Details of all four ante situm resections are shown in Table 3. All four patients underwent major hepatectomy and resection of the vena cava with reinsertion of the hepatic veins. Negative resection margins (R0) were detected in all four patients and only one had major postoperative complications. An example is shown in Figure 1.

### 3.4. Outcome and Complications

The overall complication rate and CCI were highest in the group with complex liver resections (Table 4). This applies to bile leakage, postoperative bleeding, and infections, but not to PHLF. Perioperative mortality was significantly different, but comparable between the complex (10%) and exploration group (7%). The 90-day mortality rate was highest in the exploration group (25%), compared to the complex group (14%). Perioperative and 90-day mortality was low for conventional liver resections (0% and 1%).

### 3.5. Survival

**Figure 2:**
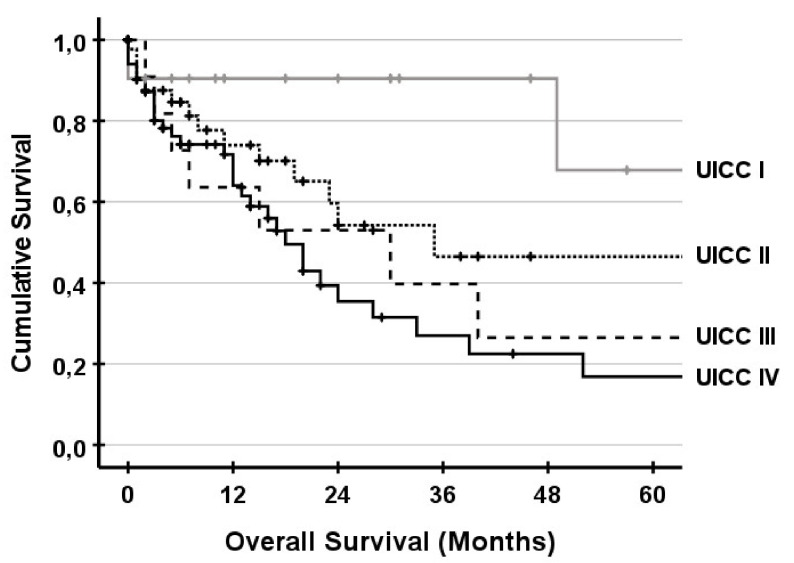
Survival for UICC stages including all 143 patients. Significance was only seen for UICC stage I compared to stage III (*p* = 0.031) and stage IV (*p* = 0.002). Five-year survival for UICC stage I, stage II, stage III and stage IV was 68%, 47%, 27%, and 17%, respectively.

**Figure 3:**
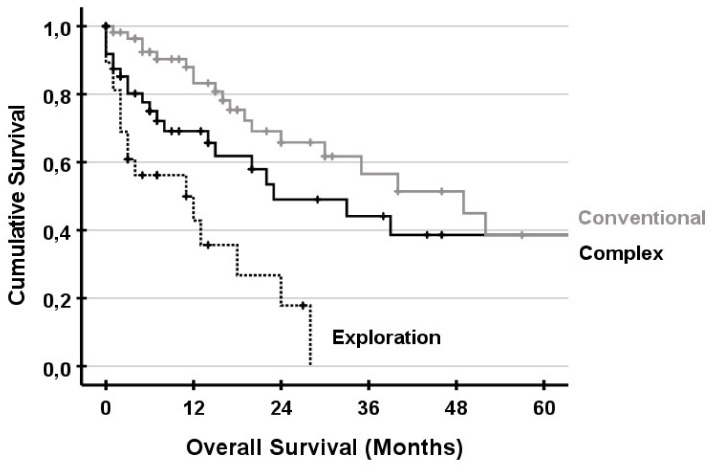
Overall survival for patients undergoing complex (**black**) or conventional (**grey**) liver resection. Exploration (**dashed line**) indicates patients who were not resectable. For patients receiving a complex liver resection, survival was significantly different compared to the exploration group (*p* = 0.009), but not to the conventional group (*p* = 0.129). Survival was also significant (*p* < 0.001) between the conventional group and exploration.

**Figure 4:**
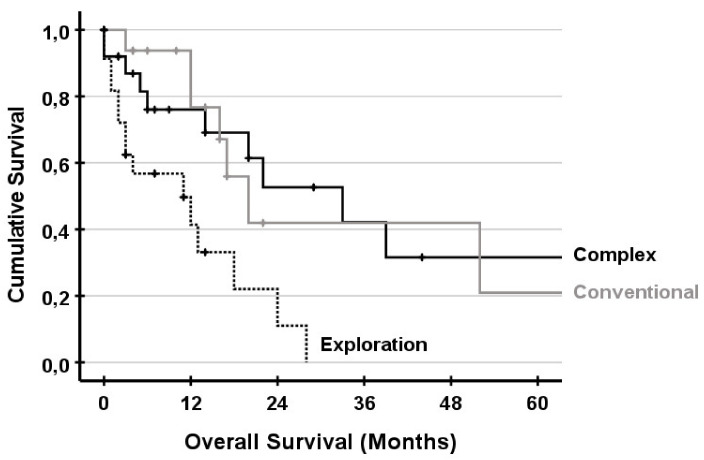
Survival for patient with UICC stage IV only. No statistical difference between complex and conventional resection was observed (*p* = 0.771). The survival of exploration was significantly different compared to complex (*p* = 0.005) and conventional (*p* = 0.007).

Overall survival for the UICC stages is shown in Figure 2. There was no significance between UICC stages II, III and IV. UICC stage I was significantly different compared to UICC stage III and stage IV. Survival for complex and conventional resections and exploration is shown in Figure 3. There was no significant difference between patients undergoing complex liver resections compared to conventional resections. Survival for non-resectable patients was significantly lower compared to complex and conventional resections. In subgroup analysis with UICC stage IV, survival was comparable and not significantly different (Figure 4).

Multivariate analysis for independent risk factors revealed resection margin and UICC stages as risk factors for overall survival (Table 5). The use of complex resections was no risk factor for long-term survival.

## 4. Discussion

Liver resection is the only curative treatment option for iCCA, and therefore, major efforts should be made to achieve tumor resection [9,10,12]. ICCAs are frequently centrally localized and often infiltrate portal and hepatic veins. Therefore, to achieve complete tumor removal, complex liver resections with vascular reconstructions including two-stage procedures are often necessary. In some circumstances, only ante situm resection represents the sole surgical option.

It is well-known that complex liver resections are generally associated with an increased morbidity and mortality [12,36,37]. This was clearly observed in the present study. The CCI with a median of 29.6 was significantly higher in the group after complex liver resections compared to the group after conventional liver resections with a CCI of 8.7. The documented mortality rate of 10% after complex liver resections with vascular reconstructions including ALPPS is high but lies within the published range and reflects the complexity of the procedure [22,23]. In a recent multicenter study of 270 patients with vascular resection in combination with liver resection for iCCA, Conci et al. reported a mortality rate of 6.7% for patients after portal vein resection and 12.5% after vena cava resection [23]. Reames et al. reported a 90-day mortality rate of 7% after 128 liver resections with major vascular resection for iCCA in a large multi-institutional analysis [22]. PHLF represents one main reason for postoperative morbidity and mortality. In the present study, the rate was 10% in the group after complex liver resections. Generally, a small FLR is mainly the cause for PHLF. Various hypertrophy concepts such as PVE or ALPPS are available to increase the FLR. However, the use of ALPPS for iCCA has been a matter of debate since its introduction [38]. Recently, Li et al. could show in a group of 102 patients with advanced iCCA from the ALPPS registry that the initially high rates of morbidity and mortality decreased steadily to a 29% severe complication rate and 7% 90-day mortality in the last 2 years [28]. Furthermore, Li et al. reported a high efficacy of 85% in achieving R0 resections. However, they only have seen an overall survival benefit for ALPPS in patients with a single lesion, not in patients with multiple lesions and, therefore, recommend ALPPS for this group [28].

A novel procedure to increase FLR is hepatic vein embolization in combination with PVE [39], which shows promising results for FLR hypertrophy compared to ALPPS [39,40,41]. Even for extended resection, the embolization of the right and middle hepatic vein is described [42]. The role of hepatic vein embolization for iCCA needs to be investigated, but seems to be a promising tool for future resection strategies [43].

It is noteworthy that we had a 0% mortality rate after our ante situm resections. The experience with ante situm resections for iCCA is limited; mostly case reports or case series are available [17,19,21,44,45]. Without doubt, the surgical procedure is challenging due to the use of an extracorporeal bypass, in situ cold perfusion and complex vascular reconstruction of the IVC and hepatic veins. However, ante situm resection offers a reasonable chance of good, long-term outcome. One may speculate that in some circumstances, ante situm or in situ resections after cold perfusion with the aim of parenchymal-sparing might be superior to complicated long-lasting two-stage procedures.

Patients with unresectable tumors have a poor prognosis [10]. During exploration, 30% of the patients that were preoperatively considered as resectable were found to be unresectable [11]. In our cohort, the survival of the exploration group was 50% at 12 months and 0% at 28 months. Surprisingly, a high 90-day mortality rate of 25% was observed in this group. This is most likely due to the fast tumor progression, which highlights the aggressiveness of iCCA. In addition, for large central iCCA, reconstruction of the bile duct is necessary, because of liver hilum involvement. In those patients, a differentiation between iCCA or perihilar cholangiocarcinoma is often challenging.

In the current study, we found no significant difference in overall survival between patients after complex and after conventional liver resections. Survival in our study depends more on resection margin status and UICC staging and not on the complexity of the resection. This is in accordance with the published data [9,10]. High UICC staging, large or multifocal tumors and vascular invasion are reported to be negative prognostic parameters in iCCA patients [12,16,46,47].

In conclusion, the effort of complex resections, such as ante situm, ALPPS, and extended resections with reconstructions of one or several hepatic vessels, is justified and results in favorable long-term outcome. Overall survival in iCCA seems to be affected by UICC stage and resections margins and not by the complexity of the surgical procedure.

## Figures and Tables

**Table 1:** Patient Demographics.

	Complex (*n* = 49)		Conventional (*n* = 66)		Exploration (*n* = 28)		
Females, *n* (%)	23	(47)	36	(55)	11	(39)	0.377
Age, median (IQR)	70	(60–75)	66	(60–73)	66	(57–75)	0.326
BMI, median (IQR)	25.9	(22.9–29.5)	24.8	(22.5–28.7)	25.7	(23.9–29.1)	0.931
CKD, *n* (%)	6	(12)	4	(6)	4	(14)	0.365
CHD, *n* (%)	3	(6)	3	(5)	4	(14)	0.228
Hypertension, *n* (%)	68	(53)	25	(38)	13	(46)	0.264
Neoadj. Chemo, *n* (%)	6	(12)	4	(6)	4	(14)	0.365
Charlson Com. Index, median (IQR)	5	(4–6)	5	(4–6)	7	(4–8)	0.582
**UICC, *n* (%)**							
Stage I	5	(10)	15	(23)	1	(4)	0.031
Stage II	13	(27)	27	(41)	4	(14)	0.028
Stage III	6	(12)	5	(8)	0	(0)	0.152
Stage IV	25	(51)	19	(29)	23	(82)	<0.001

**Table 2:** Operative details.

	Complex (*n* = 49)		Conventional (*n* = 66)		Exploration (*n* = 28)		
Operation Time, median (IQR)	346	(215–429)	210	(161–280)	132	(93–165)	<0.001
Hepaticojejunostomy, *n* (%)	16	(33)	6	(9)	3	(11)	0.003
Intraop. Transfusions, *n* (%)	22	(47)	7	(11)	2	(7)	<0.001
Blood loss, median (IQR)	1000	(0–1500)	0	(0–500)	0	(0–0)	<0.001
Pringle-Maneuver, *n* (%)	10	(21)	15	(23)	0	(0)	0.022
**Resection Margin**							
R0	31	(63)	57	(86)	n/a		0.004
R1	18	(37)	9	(14)	n/a		-
**Lymph Node Status**							
No lymphadenectomy	0	(0)	11	(17)	n/a		0.011
Negative	32	(68)	40	(61)	n/a		-
Positive	15	(32)	15	(23)	n/a		-

**Table 3:** Ante situm resections for intrahepatic cholangiocarcinoma.

No.	Age	UICC	Hepatectomy/Bypass	Type of Vascular Reconstruction	OR Time (min)	EKs (*n*)	Highest Complication /CCI	PHLF /LOS (days)	Resection Margin	Survival Status (Month)
1	29	IVa	Right Trisectionectomy/Femoral-Axillary Bypass/Portal Perfusion with HTK	Resection IVC and reconstruction with interposition of graft reinsertion of left hepatic vein	624	8	Grade I /8.7	No /16	R0	Dead (33 Months)
2	48	IVa	Left Trisectionectomy/Femoral-Axillary Bypass/Portal Perfusion with HTK	Resection of IVC with Goretex graft, reconstruction of right posterior and right anterior vein with pericardial interposition portal-vein reconstruction end-to-end	564	0	Grade II /20.9	No /27	R0	Alive (95 Months)
3	60	I	Left Hemi-hepatectomy + Seg. 1/Femoral-Axillary Bypass/Portal Perfusion with HTK	Resection of IVC with Goretex graft, reinsertion of right hepatic vein	495	5	Grade VIa /69.8	No /69	R0	Alive (10 Months)
4	55	IVa	Right Trisectionectomy/Femoral-Axillary Bypass/Portal Perfusion with HTK (Figure 1)	Resection of IVC with Goretex graft, reinsertion of left hepatic vein, portal-vein reconstruction end-to-end	470	8	Grade I /8.7	No /17	R0	Alive (8 Months)

Abbreviations: Union internationale contre le cancer (UICC); histidine-tryptophan-ketoglutarate (HTK); inferior vena cava (IVC); Comprehensive Complication Index (CCI); length of stay (LOS).

**Table 4:** Postoperative outcome and complications.

	Complex (*n* = 49)		Conventional (*n* = 66)		Exploration (*n* = 28)		
**Complications, *n* (%)**							
None	7	(14)	30	(46)	19	(68)	<0.001
Minor	20	(41)	29	(44)	4	(14)	-
Major	17	(35)	7	(11)	3	(11)	-
In-hospital death	5	(10)	0	(0)	2	(7)	0.036
CCI, median (IQR)	29.6	(20.9–42.6)	8.7	(0–24.2)	0	(0–20.9)	<0.001
Bile Leakage, *n* (%)	12	(25)	8	(12)	0	(0)	0.010
Bleeding, *n* (%)	14	(29)	7	(11)	3	(11)	0.025
Infectious, *n* (%)	11	(22)	12	(18)	0	(0)	0.029
PHLF, *n* (%)	5	(10)	4	(6)	2	(7)	0.707
Hospital Stay, median (IQR)	17	(13–24)	8	(7–12)	8	(6–11)	<0.001
90-day Mortality, *n* (%)	7	(14)	1	(1)	7	(25)	0.002

**Table 5:** Multivariate analysis for survival.

Parameter	HR (95% CI)	*p* Value
**Gender**		
Male	Ref	
Female	1.074 (0.618–1.868)	0.800
**Neoadjuvant Chemotherapy**		
Yes	Ref	
No	1.307 (0.567–3.011)	0.530
**Resection**		
Conventional	Ref	
Complex	1.219 (0.643–2.309)	0.544
Exploration	1.873 (0.775–4.525)	0.163
**Resection Margin**		
R0	Ref	
No R0	2.964 (1.638–5.363)	<0.001
**UICC**		
Stage I	Ref	
Stage II	4.160 (1.171–14.780)	0.028
Stage III	4.335 (1.107–16.986)	0.035
Stage IV	5.329 (1.604–17.705)	0.006

## Data Availability

Not applicable.
